# The Fruits of a Falsity

**Published:** 1856-10

**Authors:** 


### THE FRUITS OF A FALSITY.

An editorial of a Wall-street Daily, has been extensively published as an "Advertisement," conveying the impression, that several of the "editors and proprietors of the New York press" were among the "*remarkable cures*" of consumption, by Medicated Inhalation. To make the statement more specific, the family names of these persons were given.

The editor of the "*American Medical Gazette*" for September, declares, as to one of the names mentioned, that the whole statement was untrue, as the person alluded to, to wit, the editor and proprietor of the Commercial Advertiser, had been his own patient for many years, had never had consumption, nor had ever employed the Inhalationist referred to.

A person replies to the editor in the form of an "advertisement," in terms, not appropriate to this Journal, but the pith of the statement is, that in the latter part of 1853, he had an attack of *pleurisy.* After medical advice here, he went South, remained a month and returned hopeless of a cure, having "a racking cough;" "profuse night sweats;" "very bad expectoration, streaked with blood;" "losing flesh and no appetite." In February, he began the Inhalation treatment, eventually got well, and remains so to the present hour.

Every educated physician will recognize this as a description of the rise, progress, and termination, of a case of common pleurisy. And all controversy could easily have been avoided, if a plain straightforward statement had been made of the simple facts of the case, to the following effect:

"I, John Smith, formerly a proprietor in the job department of the Commercial Advertiser, hereby declare, that in 1853, I had an attack of pleurisy, and failing to be cured in two or three months, employed Medicated Inhalation, eventually got well, and remain so at the end of two years."

This would have been plain sailing, and would have misled no one, as no very important fact was communicated; for after all, it amounted simply to this:

"I had pleurisy, got well in three or four months, while Inhaling, and remain so."

But there is nothing remarkable here. Quite a number of persons get well of pleurisy, without doing any thing at all. Multitudes are cured of pleurisy, annually, all over the globe, by the very commonest physicians. We do not exactly see the use of publishing the cure of a case of pleurisy. It rather indicates that the Inhalationists are greedy of a cure of any thing at all, by their system. Elated to the very skies, because a man got well of pleurisy while inhaling, it being just about the time that a "pretty good case of pleurisy" ordinarily gets well of itself, especially with the aid of approaching warm weather, as in this instance.

But it is very clear, that the intended effect of the "advertisement" of this editorial "*Affidavy*," was to produce an impression, that a New Yorker, of character and standing, being one of the editors and proprietors of the Commercial Advertiser, had been cured of consumption, by employing Medicated Inhalation. This was the idea which the veteran editor of the *Medical Gazette* declared to be wholly untrue, and *all* the parties know it to be wholly untrue.

But as few men can calmly bear being placed in a false position, especially, when rightly done, a person, claiming to be the *brother* of one of the editors and proprietors of the Commercial Advertiser, exclaims:

"Whereas, it is true, I never was one of the editors of the Commercial Advertiser; but my brother is, and although I never was one of the proprietors of the Commercial Advertiser,

I was once one of the proprietors of its job department. And if I never had consumption, I had pleurisy, and got well of it, while employing Inhalation—and whereas, I, who am not, nor ever have been, one of the editors and proprietors of the New York press, but was once one of the proprietors of the job department of one of them, and got well of pleurisy while employing Medicated Inhalation—*therefore*, I am one of the editors and proprietors of the New York press, and am a case, (very true,) among "the remarkable cures" of consumption, by means of Medicated Inhalation."

*Does any true system require machinery like this?*

